# Echocardiographic quantification of myocardial function using tissue deformation imaging, a guide to image acquisition and analysis using tissue Doppler and speckle tracking

**DOI:** 10.1186/1476-7120-5-27

**Published:** 2007-08-30

**Authors:** Arco J Teske, Bart WL De Boeck, Paul G Melman, Gertjan T Sieswerda, Pieter A Doevendans, Maarten JM Cramer

**Affiliations:** 1 Department of Cardiology, University Medical Centre Utrecht, The Netherlands; 2 Department of Cardiology, St. Elisabeth Hospital Tilburg, The Netherlands

## Abstract

Recent developments in the field of echocardiography have allowed the cardiologist to objectively quantify regional and global myocardial function. Regional deformation (strain) and deformation rate (strain-rate) can be calculated non-invasively in both the left and right ventricle, providing information on regional (dys-)function in a variety of clinical settings. Although this promising novel technique is increasingly applied in clinical and preclinical research, knowledge about the principles, limitations and technical issues of this technique is mandatory for reliable results and for implementation both in the clinical as well as the scientific field.

In this article, we aim to explain the fundamental concepts and potential clinical applicability of strain and strain-rate for both tissue Doppler imaging (TDI) derived and speckle tracking (2D-strain) derived deformation imaging. In addition, a step-by-step approach to image acquisition and post processing is proposed. Finally, clinical examples of deformation imaging in hypertrophic cardiomyopathy (HCM), cardiac resynchronization therapy (CRT) and arrhythmogenic right ventricular dysplasia/cardiomyopathy (ARVD/C) are presented.

## Background

There is an increasing need for diagnostic modalities able to objectively quantify myocardial function. Quantification of regional myocardial function with ultrasound is challenging on its own. Visual assessment of wall motion and thickening requires extensive training[1] and remains highly subjective[2]. In addition to this visual assessment on 2-dimensional B-mode images and new 3-dimensional recordings, "classic" M-mode echocardiography can be used to evaluate myocardial thickening. Unfortunately, M-mode can only be used in a limited number of segments since it has to be perpendicular to the investigated myocardial segment. New techniques such as anatomical M-mode have partly overcome the problems with insonation angle, at the expense of a reduced temporal resolution. Moreover, this method only provides uni-dimensional semi-quantitative assessment of myocardial thickening/thinning without information on longitudinal or circumferential function. Tissue velocity imaging along the long axis of the ventricle is a commonly used and well validated technique to quantify longitudinal ventricular function. Tissue velocities at the ventricular base represent the integral of myocardial shortening velocity from base to apex and therefore provide information on global, rather than on regional ventricular function.

Tissue deformation imaging is a recently introduced technique which enables the objective assessment of regional myocardial deformation assessed by ultrasound based strain and strain rate using myocardial Doppler data or B-mode images. This is a promising new technique to quantify regional left and right ventricular function and appears of added value in unmasking or unraveling cardiac pathology (table 1). Knowledge of its principles and limitations is mandatory for proper application and reliable interpretation of the results both in the clinical as well as the scientific setting. This review therefore aims to explain the basic concepts and potential clinical applicability of strain (ε) and strain-rate (SR) for both tissue Doppler derived and speckle tracking derived deformation imaging. In addition, a step-by-step approach of image acquisition and post processing will be discussed.

**Table 1:** The additional value of tissue deformation imaging in the field of echocardiography

**Additional value**	**Potential utilities**
Unmasking subtle pathology	Early diagnosis coronary artery disease [28] and cardiomyopathy [23]
Quantifying myocardial function	Objective assessment of regional function [29]
Visualizing timing issues	Quantification of timing within a heart cycle [30]
Detecting subtle changes over time	Therapy evaluation in patient follow-up [31]
Improving inter/intra observer variability	Increasing accuracy of stress echocardiography [32]

## Myocardial deformation

Following electro-mechanical activation, the myocardium deforms during systole due to sarcomere shortening. This active deformation causes a reduction in intracavitary size, resulting in the ejection of blood from the ventricle. In diastole the original ventricular geometry is restored due to active relaxation and passive filling following atrial contraction. Since myocardial tissue is virtually incompressible, the volume of the ventricular wall remains the same during the cardiac cycle and, thus, deforms in three dimensions. During systole there is three-dimensional deformation which can be expressed in three ventricular coordinates: a longitudinal shortening and a circumferential shortening and a radial thickening.

**Figure 1:**
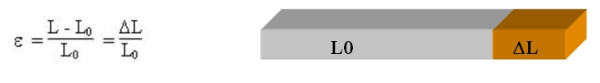
**Calculation of deformation**. Calculation of strain (ε), with L_0 _as the original length (grey bar in figure), ΔL the change in length (orange bar) and L as the total length (grey and orange bar).

**Figure 2:**
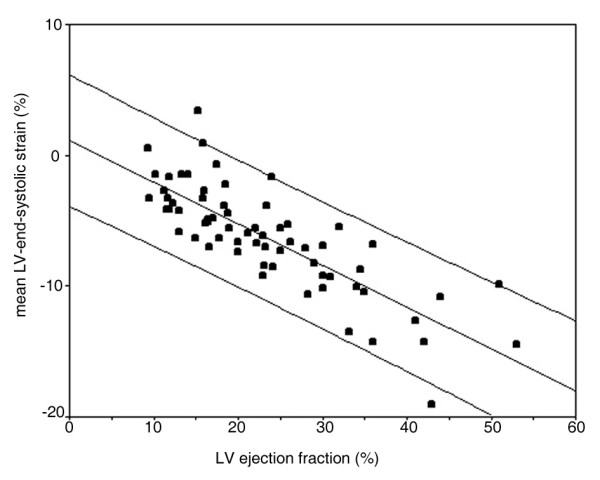
**LVEF – global 4Ch strain correlation**. Correlation plot of absolute strain values vs LV ejection fraction (LVEF) in a CRT population (unpublished data, De Boeck, et al). Absolute strain values are calculated as mean of the end systolic (global) strain values of the septal and lateral wall using 2DSE. LVEF is calculated using the Simpson's rule. Note the good correlation over a broad range of LVEF.

**Figure 3:**
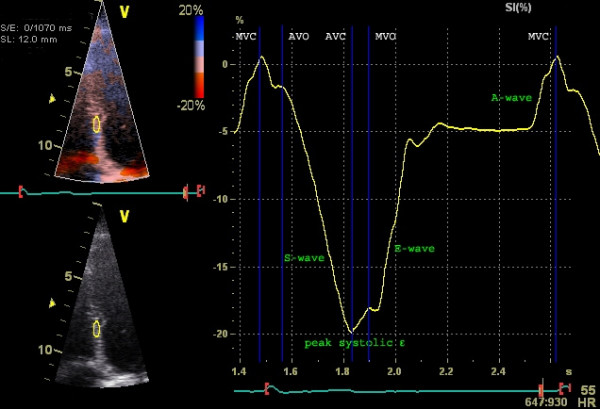
**Example of longitudinal strain graph (Doppler derived)**. Longitudinal strain derived from tissue Doppler data in the interventricular septum in a healthy person. Y-axis represents strain (%), X-axis represents time (one cardiac cycle). Blue lines represent cardiac events: (MVC) mitral valve closure and (MVO) opening, and (AVC) aortic valve opening and (AVC) closure.

Myocardial deformation is expressed as a one dimensional parameter: strain (ε). It defines the total deformation during the cardiac cycle relative to the initial length at the onset of the cardiac cycle, and is expressed in percentages (figure 1). This implies that longitudinal and circumferential (systolic) shortening result in a negative strain and radial (systolic) thickening in a positive strain. When the initial length of the investigated myocardial segment is known during the cardiac cycle, the relative length change (strain) can be calculated throughout the entire cardiac cycle. The local end-systolic strain value reflects the regional ejection fraction and the global LV end-systolic strain reflects the LV ejection fraction [3] (Figure 2). When visualized in a graph (figure 3) the different phases of the cardiac cycle can usually be identified: during systole the strain values become more negative (S-wave) with the negative peak at the aortic valve closure, representing the maximal longitudinal myocardial shortening during contraction (or peak systolic strain). In diastole the strain values return towards zero (towards the original length of the analyzed myocardial segment at the onset of the cardiac cycle) in three phases: (1) the early, or rapid filling phase (E-wave) followed by (2) a plateau phase, or diastasis and finally (3) atrial filling (A-wave).

**Figure 4:**
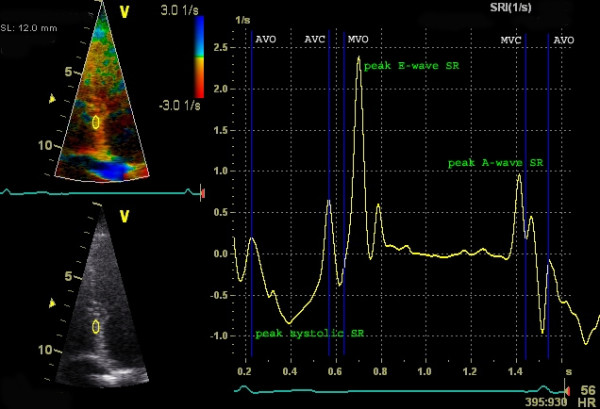
**Example of longitudinal strain-rate graph (Doppler derived)**. Longitudinal strain-rate using the region of interest of figure 3. Y-axis represents strain-rate (s^-1^), X-axis represents time (one cardiac cycle). Note the negative SR during systole when the ε becomes more negative (myocardial shortening) in figure 3 and the positive SR during the upstroke of the strain-curve during the E and A-wave. The SR is zero when no deformation occurs.

The speed at which the myocardial deformation occurs is the strain-rate (SR) and is expressed in s^-1^. SR depicts the change in strain over a period of time. Thus, when the myocardium shortens there is a negative SR and, the steeper the slope of the strain-curve, the higher the SR-values. The peak systolic strain rate correlates well to loading independent indices of contractility and hence provides valuable information on regional contractile function [3,4]. Comparable to strain, when plotted in a graph (figure 4) the different cardiac phases can be recognized: in systole a negative deflection indicating myocardial shortening with a peak systolic SR at the steepest part of the strain-curve, and in diastole two positive deflections: the E- and A-wave and diastasis when no change in strain occurs.

To optimally assess regional myocardial function, both strain and SR need to be calculated since they provide complementary information: end systolic strain estimates ejection fraction and peak systolic SR is a measure of contractility. By analogy, when driving a car both the total distance of the journey as well as the speed of the car during the journey provide valuable information.

## Tissue deformation imaging

Currently there are two different methods to calculate myocardial deformation using ultrasound: tissue Doppler derived strain and two-dimensional strain derived from B-mode images (speckle tracking). These two techniques will be briefly explained in the following section. Notably, differences in software packages may exist between manufacturers [5]. The examples in this review are based on the commercially available software packages from GE Vingmed (GE Vingmed ultrasound, Horten, Norway).

### Tissue Doppler strain

#### Principles

**Figure 5:**
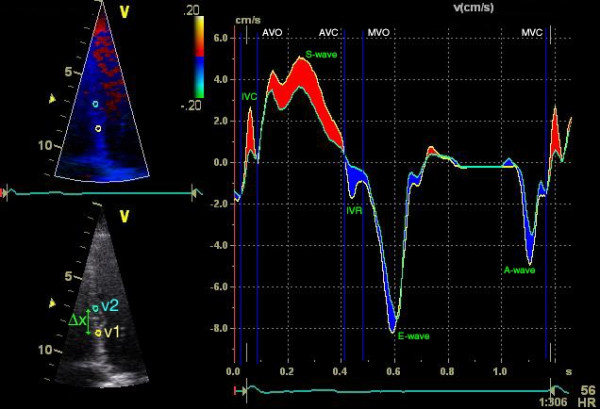
**Calculation of strain-rate using the velocity gradient**. Longitudinal velocities using two ROI points in the same recording as figure 3. Y-axis represents velocity (cm/s), X-axis represents time (one cardiac cycle). IVC = isovolumic contraction; IVR = isovolumic relaxation. Calculation of strain-rate (SR) can be derived from the spatial velocity gradient (Δv) at a defined distance (Δx). The red area indicates a negative Δv due to shortening, resulting in a negative SR (and thus a reduction in strain) and the blue area results in a positive SR. This is demonstrated in figure 4.

By color coded tissue Doppler images (TDI), the velocity relative to the transducer is recorded and can be calculated in each pixel (figure 5). In the longitudinal recordings, the gradual increase in velocity from the nearly stationary apex to a maximal velocity at the annular plane is called the velocity gradient and is generated by longitudinal shortening of the myocardium. Within a defined region of interest (ROI) the difference in velocities is used to calculate the SR using the velocity of the distal (v1) and the proximal (v2) point within the ROI and the distance between these two points (see formula, figure 4). Strain is calculated from the SR values by temporal integration.

SR=v1−v2Δx=ΔvΔx
 MathType@MTEF@5@5@+=feaafiart1ev1aaatCvAUfKttLearuWrP9MDH5MBPbIqV92AaeXatLxBI9gBaebbnrfifHhDYfgasaacH8akY=wiFfYdH8Gipec8Eeeu0xXdbba9frFj0=OqFfea0dXdd9vqai=hGuQ8kuc9pgc9s8qqaq=dirpe0xb9q8qiLsFr0=vr0=vr0dc8meaabaqaciaacaGaaeqabaqabeGadaaakeaacqWGtbWucqWGsbGucqGH9aqpdaWcaaqaaiabdAha2naaBaaaleaacqaIXaqmaeqaaOGaeyOeI0IaemODay3aaSbaaSqaaiabikdaYaqabaaakeaacqqHuoarcqWG4baEaaGaeyypa0ZaaSaaaeaacqqHuoarcqWG2bGDaeaacqqHuoarcqWG4baEaaaaaa@3FF2@

From this formula it is also clear that if a myocardial segment is not deforming during systole, for example due to an infarction, there is no difference in velocities and thus a SR of zero, although the segment could show a displacement and velocity due to tethering of a functional neighboring segment. This translated velocity on the non-deforming segment could give the impression of normal function when assessing it by velocity imaging only, but this is unmasked when calculating the regional SR and ε.

TDI is one-dimensional, thus only deformation along the ultrasound beam can be derived from velocity. Therefore, only the longitudinal SR/ε in the apical views and radial SR/ε in the parasternal views can be calculated.

#### Validation

Several studies have established the robustness and potential useful role of this novel echocardiographic technique.

The accuracy of the measurement of velocities has recently been investigated using tissue mimicking gelatin blocks in two commercially available ultrasound systems [5]. This *in-vitro *study reported a very high degree of accuracy for velocity measurements, although there was a small overestimation of ε and SR[5]. *In-vivo *invasive studies, using sonomicrometry as a reference method, have confirmed the validity to calculate strain and strain-rate from tissue velocity over a wide range of strains in animal models [6,7]. Nowadays, the reference method for non-invasive validation in humans is three-dimensional tagged magnetic resonance imaging, which relies on deformation of tag lines to assess strain in 3 dimensions. Doppler derived strain measurements in humans, both healthy controls and patients with acute myocardial infarction, under different conditions (at rest and during Dobutamine infusion) have proven to be accurate when comparing ultrasound with MRI [8].

Thus, tissue Doppler imaging is a well validated technique to calculate strain and SR over a wide range of conditions, indicating the feasibility for the usefulness of this technique in the clinical setting.

#### Limitations

When evaluating regional myocardial function with tissue Doppler derived SR/ε, knowledge of the limitations of this technique is essential to ensure appropriate acquisition as well as correct post-processing since artifacts often mimic pathology. The most important and common artifacts will be briefly discussed in this section.

**Figure 6:**
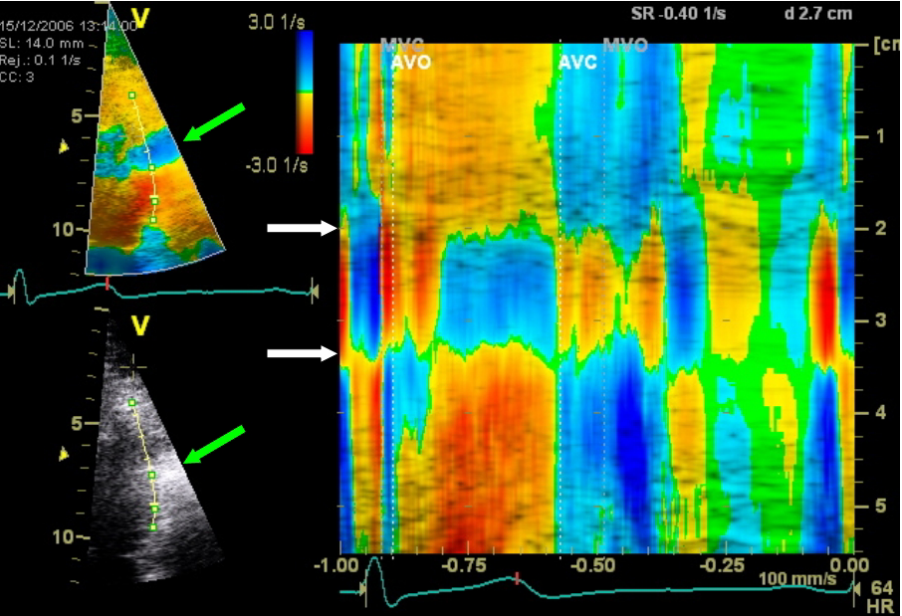
**Impact of a stationary artifact on Doppler derived SR**. TDI derived color coded M-mode of strain-rate in the lateral wall of one cardiac cycle. A stationary artifact (between white arrows) disturbs the velocity derived parameters. Note the reversal of the strain rate in the artifact and the overestimated SR below the artifact. This pattern closely resembles pathology, although the reversal in each phase (also during the atrial contraction) suggest an artifact which is also clearly seen on the B-mode images in this case (green arrows). Bottom = base; top = apex; yellow-red = negative SR; green = no SR; Blue = positive SR.

Since all calculations are derived from the measured velocities, it is essential to work with a data set which provides the true velocities of the myocardium. Reverberations, side lobes and drop out all interfere with obtaining the true velocities and disturb the data used to calculate SR and strain. Reverberations and side lobes are stationary artifacts of extracardiac structures projecting through the myocardium. The measured velocity within such an artifact is close to zero, resulting in 1) random noise within the artifact 2) an overestimation of negative SR/ε below the artifact, 3) a reversal directly above the artifact and 4) an underestimation above the reversal region (Figure 6). If, for example, the true v_1 _is 4 cm/s and v_2 _is 3 cm/s, but due to a stationary artifact the measured v_2 _is 0 cm/s. This means that the calculated Δv is 4 cm/s instead of the true 1 cm/s, overestimating the SR (see formula) and strain. Drop-out interferes in a similar manner in the calculation of SR, since no velocities are measured, within the drop out zone SR is close to zero, giving the impression of hypo- or akinesia.

Deviation of the insonation angle is another important limitation of the technique. If the ultrasound beam deviates from the direction the investigated myocardial segment moves, the measured velocity (v_m_) is lower than the actual myocardial velocity. This underestimation is dependent on the angle between the myocardial wall and the ultrasound beam, this implies that v_m _reduces if the angle increases. Angle correction however, is not an option. As explained earlier, the myocardium deforms in three dimensions, meaning that when measuring longitudinal deformation, one has to keep in mind the simultaneous radial deformation. When the ultrasound beam is parallel to the myocardial wall, the v_m _is the actual velocity and the v_m _of the transverse deformation is zero, since the movement is perpendicular to the ultrasound beam. When the angle changes, the contribution of the transverse velocity increases in the v_m_. For the resulting SR/ε this results in a combination of both a negative (longitudinal) and a positive (transverse) strain, severely disturbing the derived SR/ε patterns. For acceptable calculations, an angle deviation below the 20 to 15 degrees is mandatory.

#### Image acquisition and post-processing

As pointed out, TDI is a technique which has some limitations inherent to the technique, which could potentially disturb the derived parameters. Optimal image acquisition and knowledge of post-processing techniques are essential to minimize artifacts and, respectively, deal with them in post processing.

##### Image acquisition

The ultrasound investigation starts with an optimal ECG signal with a clear definition of the QRS-complex and P-wave ensuring a consistent ECG-triggering. Pulsed or continuous wave measurements of the valves are needed to incorporate event timing in the off-line measurements. An optimal quality in the 2D gray scale image is mandatory for a high quality tissue Doppler recording. In the following section we present a step-by-step approach for image acquisition.

1) The myocardial wall needs to be optimally visualized with a clear delineation of myocardial tissue and extracardiac structures (e.g. suppression of the blood pool), a minimum of extracardial artifacts should be achieved and the angle of interrogation limited (deviation below 15–20 degrees). The latter is achieved by orienting the transducer parallel or perpendicular to the investigated wall.

2) TDI settings need to be adjusted for optimal post-processing. The velocity scale needs to be adjusted to avoid aliasing. Although a reduction in the velocity scale will result in lower frame rates, the lowest possible velocity scale will result in the highest spatial and temporal resolution combination. Further optimization of the temporal and spatial resolution is achieved by narrowing of the image sector until it is slightly wider than the investigated wall or wall segment. Wall by wall acquisition is essential to achieve adequate frame rates, for reliable SR calculations the optimal sampling rate is >180 frames/sec.

3) Three complete cardiac cycles are recorded digitally during breath hold (to minimize image translation) in the presence of a regular rhythm. This will allow filtering of average noise during post processing without losing resolution.

Longitudinal ε/SR can be derived from the apical three (posterior and anteroseptal wall), two (inferior and anterior wall) and four chamber views (lateral, septal and RV free wall). Radial strain can be derived from the parasternal recordings of the posterior wall. The measurement of septal wall radial strain often produces artifacts due to the morphological and functionally bilayered architecture (LV and RV fibers) of the interventricular septal wall [9]. Circumferential strain can be calculated from the short axis recording (lateral and inferoseptal wall).

##### Post-processing

Knowledge and experience in the post-processing techniques are important to interpret the ε and SR graphs and to distinguish pathological deformation from artifacts.  All commercial software programs are equipped with numerous settings to offer the possibility to optimize the calculations to derive SR from velocity. These settings are available to reduce the 'random-noise' in the SR and ε graphs. With the reduction of noise, the SR and ε graphs are smoothed with the consequence that potentially important temporal or regional information is lost. Therefore, a maximal signal to noise ratio should be achieved with a maximal spatial resolution. Averaging more than one cardiac cycle (reduction of random noise and the beat-to-beat variation), temporal (reducing the effective sampling rate) and spatial averaging and increasing the offset length (changing the Δx in figure 1) are alternative options to reduce noise, although, with careful tracking this is often unnecessary using default settings. Another important setting is the correction for drift in the strain graphs. Assuming that the length of the myocardium returns to its original length at the end of the cycle, the strain value should return to zero. Time integrating the strain-rate can result in drifting of the strain curve. Drift compensation could introduce an error in the strain estimates and should be taken into account when interpreting the strain graphs.

All derived parameters are calculated within a specific region of interest (ROI), therefore the ROI needs to follow myocardial motion during the cardiac cycle. A brief explanation on tracking is described in the following segment.

1) The ROI should be defined; this implies adjustment of both the offset length and ROI size. These parameters can vary and depend on the investigated parameter (radial vs. longitudinal strain) or the clinical question (since small defects appear normal in a large ROI or offset length).

2) The ROI should be placed in the myocardium, typically, three ROI locations are defined in each wall (basal-, mid- and apical segment).

3) The ROI should be tracked throughout the cardiac cycle to follow the myocardial movement. Special attention should be made to avoid stationary artifacts (e.g. reverberations) and the blood pool (see Additional File 1).

4) Finally the quality of the tracking should be checked. Sub-optimal tracking of the ROI will result in drift in the strain-graphs and in a noisy SR-graph. The ROI position can be corrected after the identification of the noisy frames (to eliminate frame-to-frame artifact or blood-pool noise) which are best identified in the SR graph.

##### Parameters

**Figure 7:**
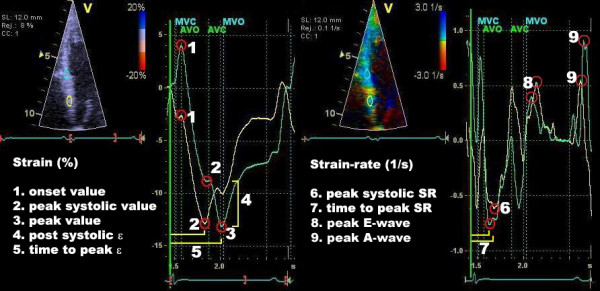
**Strain and strain-rate parameters**. *Left*: s train (ε) and *right*: strain-rate (SR) measurements of the basal and mid segment of the interventricular septum. Note the prestretch and the post systolic shortening (4) in the cyan curve. Post systolic strain is defined as the amount of deformation after the AVC (value 3 minus value 2). In the yellow curve, the peak systolic value and the peak value are the same, while in the cyan curve these are two distinctive points (2 and 3). From this, the post-systolic index (PSI) can be calculated: (4)/(3) × 100 = PSI (%). The definition of the onset value (1) could also be defined at the onset of the QRS-complex for the yellow curve.

Numerous measurements and calculations can be performed after the tracking of the ROI. These can be divided into parameters concerning the timing and/or the magnitude of strain and SR. The most relevant measurements are presented in figure 7. For shortening strains, the peak systolic strain value is defined as the lowest value between aortic valve opening and closure. Post-systolic strain is defined as myocardial deformation (shortening for longitudinal and lengthening for radial function) after the aortic valve closure. From these values, the post-systolic index (PSI) can be calculated (figure 7). For the SR trace, comparable parameters can be obtained as in the velocity trace; peak systolic, peak early- and late diastolic SR and time to peak systolic SR, although the last measurement can be unreliable, due to the noisy aspect of the SR curves.

### Speckle tracking/two-dimensional strain

#### Principles

**Figure 8:**
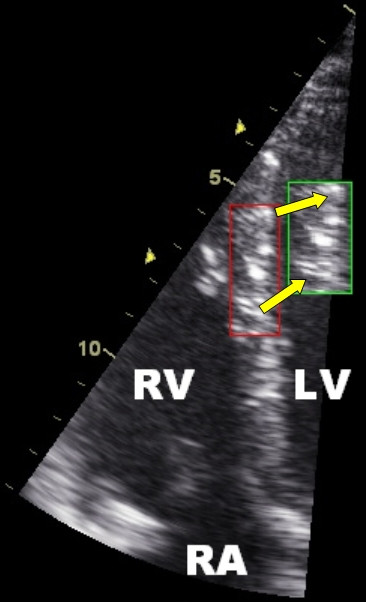
**Typical speckled pattern of the myocardium on ultrasound in the septal wall**. The tracking algorithm follows a unique speckle pattern during the cardiac cycle. The red square represents the starting location and the green square the location of the pattern at end systole. Note the change in distance between the speckles due to deformation (longitudinal shortening and radial thickening of the square). RV = right ventricle; LV = left ventricle; RA = right atrium.

2-dimensional strain imaging is a new technique which uses standard B-mode images for speckle tracking analysis, in which the speckled pattern (acoustic backscatter generated by the reflected ultrasound beam) is followed frame by frame. This speckle pattern is unique for each myocardial region and it is relatively stable throughout the cardiac cycle. The displacement of this speckled pattern is considered to follow myocardial movement and a change between speckles represents myocardial deformation (Figure 8). When tracking a defined region of speckles, a software algorithm follows the change in geometric position of this region, frame by frame, and extracts the displacement, velocity, strain and strain rate of a defined myocardial segment. In contrast to TDI derived parameters, speckle tracking is an angle independent technique as the movement of speckles can be followed in any direction. For the apical views this implies that not only longitudinal, but also transverse parameters can be calculated, which is not possible in TDI recordings. In short axis images both circumferential and radial parameters can be calculated for all myocardial segments. In addition to the circumferential deformation parameters, ventricular rotation and twist (additional parameters for LV function) can also be calculated using this technique [10,11]. Since the deformation parameters can be calculated in two dimensions (while TDI derived parameters are one-dimensional), this technique is often referred to as two-dimensional strain echocardiography (2DSE).

#### Validation

Validation of early versions of 2DSE software in a tissue mimicking gelatin block revealed a good correlation compared to sonomicrometry, although values (both strain and strain-rate) were overestimated in the lower range of values [12]. These findings were also found in an animal model (sonomicrometry in pigs with myocardial infarction), where 2DSE was found to slightly overestimate values compared to the reference test. A weaker correlation was found in the lower range of deformation values [12]. Tracking is affected by dyskinetic segments (e.g. ischemia), causing the fiber orientation to change, thus affecting acoustic properties and speckle integrity. Using a new (two-stage) tracking algorithm, a better correlation was found between the 2DSE values and those obtained using sonomicrometry over the entire broad range of tested clinical relevant values [13]. For radial and circumferential parameters, a good correlation was found using sonomicrometry in dogs [14].

The first study to determine the feasibility and accuracy of 2DSE in patients with myocardial infarction reported that of all segments, 98% in the control population and 80% in the patient group could be analyzed, findings which were reproduced in later studies [14]. Importantly, 2DSE data are highly reproducible and analysis is affected by only small intraobserver (mean 4.4 (SD 1.6)%) and interobserver variability (7.3 (SD 2.5)%) [15]. Values were significantly reduced in the infarcted segments, implying a reduction in systolic deformation. There was no significant difference between TDI and 2DSE parameters [16]. In a direct comparison of TDI and 2DSE with MRI-tagging as a reference, comparable values were found for 2DSE and TDI in both normal and dysfunctional segments. For radial measurements, 2DSE was more reliable than TDI [17].

#### Limitations

**Figure 9:**
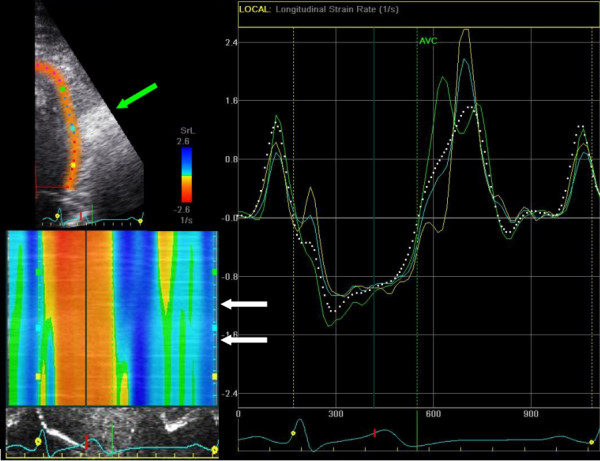
**Impact of stationary artifact on 2DSE**. 2DSE derived strain-rate of one cardiac cycle in the lateral wall in the same ultrasound examination of figure 6. *Top left*: B-mode recording with 2DSE ROI of the lateral wall. *Bottom left*: Color-coded M-mode of strain-rate values. The stationary artifact can be seen at the level of the green arrow. Due to the implementation of a clutter filter in the tracking algorithm, 2DSE is less influenced by artifacts. Note the normal SR curves (*right*) despite the prominent artifact. Consequently, no reversal of strain-rate is seen in the color-coded M-mode between the white arrows (compare to figure 6). The appropriate ROI tracking can be seen in the additional file (see Additional File 2)

2DSE is generally more smoothed and, therefore, less influenced by artifacts than TDI derived strain (Figure 9/Additional File 2), but also less sensitive to detect small regions of pathology. Some other limitations of this technique exist. Despite noise reduction by increased smoothing and despite an anti-clutter filter, drop-out is still problematic and stationary reverberations are sometimes still tracked or interfere with the frame-by-frame tracking, resulting in drift or incorrect calculation of strain and strain-rate. In addition, a clear delineation of the endocardial border is also important for a reliable radial and transverse tracking. Since spatial resolution is lower in the transverse direction, tracking perpendicular to the ultrasound beam becomes less robust.

At present, the optimal frame rate for speckle tracking seems to be 50–70 frames per second (FPS), which is lower compared to TDI (>180 FPS): this could result in undersampling, especially in patients with tachycardia. Rapid events during the cardiac cycle (e.g. isovolumic phases) may disappear all together, and peak SR and velocity values may be reduced due to under sampling, especially in isovolumic phases and in early diastole. Higher frame rates could reduce the undersampling problem, although this will result in a reduction of spatial resolution and, consequently, less optimal ROI tracking [18]. Low frame rates increase the spatial resolution, but introduce a new problem. Speckle-tracking software uses a frame-by-frame approach to follow the myocardial movement and searches each consecutive frame for a speckle pattern closely resembling and in close proximity to the reference frame. With a too low frame rate the speckle pattern could be outside the search area, again resulting in poor tracking.

**Figure 10:**
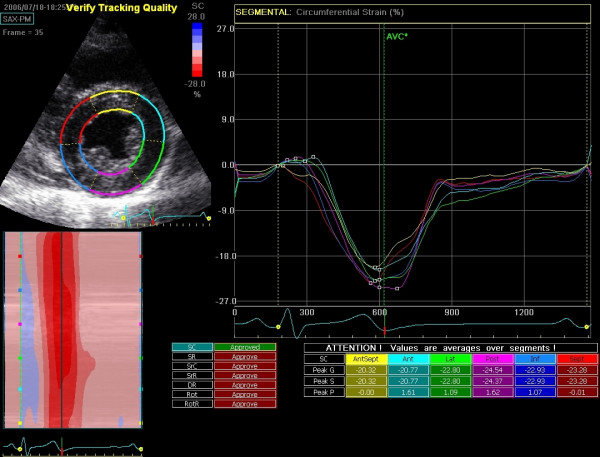
**"Results-screen" of 2DSE**. Circumferential strain is calculated on a short axis recording at the level of the papillary muscle in a healthy individual. On the top left the short axis view with the ROI divided into six segments; top right: graphical representation of the selected parameter of all accepted segments; bottom right: peak values of all segments, these values are automatically calculated and can be manually adjusted in the graph if necessary; middle bottom: all available parameters (radial and circumferential strain and strain-rate, radial displacement, rotation and rotation rate), if approved, these values are stored; bottom left: color coded M-mode of the selected parameter.

Another limitation of 2SDE is the fact that calculated parameters are averaged over the myocardial segment when using the "results page" of the software program (see figure 10). For most pathologic conditions this is not an important limitation, although in conditions with small regions of myocardial dysfunction, such as early stages of hypertrophic cardiomyopathy or arrhythmogenic right ventricular dysplasia, the averaging could result in normal deformation parameters due to normal deformation properties of the adjacent myocardium.

Optimal tracking of the ROI is not only dependent on optimal image quality, but also on the implemented tracking algorithm. Software programs designed for speckle tracking are relatively new and are subjected to periodical improvements. Implementation of a new two-stage tracking algorithm (correcting initial 'rough', block matching estimates using optical flow techniques [19]) has indeed shown to improve tracking quality [13]. However, different tracking algorithms potentially produce different results, therefore it should be kept in mind that a periodical update of the software package conceivably influences reference values.

#### Image acquisition and post-processing

##### Image acquisition

The same basic principles for image acquisition as mentioned for TDI recordings apply for 2DSE: digitalized recordings of a minimum of one cardiac cycle are made during breath hold (to minimize through plane motion) with a stable ECG recording. Since the angle of interrogation does not influence strain and strain-rate calculations, the transducer can be placed off-axis to obtain the optimal gray scale image. However, misalignment in the third dimension (foreshortening in apical views and oblique transsections in parasternal views) cannot be corrected in post-processing and should be correct. We would like to state that optimal frame rates for 2DSE are still under discussion, but in our own experience: a frame rate of 50–80 FPS seems to result in the optimal tracking quality in the left ventricle long and short axis. For single wall recordings, a frame rate between 70–110 FPS will result in proper tracking [18].

Visualization of the myocardial wall needs to be optimal, with a clear delineation of myocardial tissue and extracardiac structures and avoidance of drop-out since this will result in unacceptable ROI tracking and drift. The optimal balance between temporal and spatial resolution is achieved when adjusting the image sector slightly wider than the interrogated wall. For small-angle acquisitions this has the advantage that frame-rate can be reduced into the 50–110 FPS range while greatly enhancing the lateral resolution and overall quality of the image. Moreover, near field clutter can be reduced with the implementation of dual-focus imaging when artifacts are prominent. For parasternal recordings (for radial and circumferential function on the short axis) small angle recordings are not possible, all myocardial segments need to be in the image field. For any recording, it should be kept in mind that all myocardial segments need to be visualized optimal, since poor tracking of any segment often affects tracking of the adjacent segments.

##### Post-processing

Comparable to TDI calculations, careful post-processing is mandatory to obtain reliable regional data, although ROI tracking is less influenced by artifacts and are easier to identify as inappropriate ROI tracking. ROI tracking in 2DSE is discussed in the following section.

1) The ROI is defined by tracing the endocardium in a still frame (default at end systole).

2) The ROI width is set to match the myocardial thickness. An automated software program calculates the frame-to-frame displacements of the speckle-pattern within the ROI throughout the cardiac cycle.

3) The resulting tracking quality is then scored as either acceptable or non-acceptable. Since the program follows any speckle pattern, tracking of a stationary artifact during the cardiac cycle is not uncommon, and often scored as appropriate tracked. Therefore, the tracking has to be visually checked and adjusted if necessary. Even the slightest failure of ROI tracking can result in drifting of the calculated strain curve.

For each myocardial segment, velocity, displacement, strain and SR are calculated in two dimensions: longitudinal and transverse parameters in the apical recordings and circumferential and radial parameters in the parasternal recordings (Figure 10). For the right ventricle, only longitudinal parameters can be reliably calculated due to the thin wall. Additionally, ventricular rotation and rotation-rate are calculated for the short axis recordings. With these parameters, left ventricular torsion can be calculated. Likewise for the TDI parameters, temporal and spatial smoothing can be adjusted.

## Comparability between TDI and 2DSE

Both TDI and 2DSE derived parameters are accurate quantitative measures of local longitudinal myocardial deformation, and thus (dys)function and will general yield comparable values for local deformation and deformation rates in both the LV [16] and the RV [18]. Variability in the measurements and technical factors however, make that minor differences between the calculated parameters should be anticipated when comparing values of TDI and 2DSE in a single patient.

Most of the observed differences between the two techniques are those inherent to the limitations described earlier. For example, TDI is not possible when the investigated wall is not optimally aligned, or in the presence of a stationary artifact, whereas 2DSE is less influence by these interferences. On the other hand, 2DSE has a relative low temporal resolution hindering tracking in the presence of high heart rates where also undersampling becomes an issue [20], this is not a problem with TDI, where frame-rates are higher (up to 250 FPS).

Practical differences between the two techniques are an additional factor when choosing between one or the other. Training requirements for both image acquisition and post-processing are equal, although TDI is generally perceived as more difficult to master. This is particularly the case for post-processing. In our experience, acquisition time is comparable between the two techniques. Post-processing time, however is significantly shorter with speckle tracking [21] with approximately 2 minutes for 2DSE and 11 minutes for TDI analysis of 16 segments from 3 apical views. This mainly results from a more rapid tracking and parameter extraction in 2DSE, which is automated. The number of segments which can be analyzed with either technique is comparable (Table 2, feasibility), although some studies reported a lower feasibility for 2DSE compared to TDI [21]. The reproducibility of 2DSE is generally better in the LV than with TDI (Table 2). In the RV, this difference is not seen. Despite the advantages 2DSE offers compared to TDI, we feel that both techniques are of equal or complementary value in the quantification of myocardial function.

**Table 2:** Feasibility and reproducibility. Feasibility is expressed as a percentage of analyzable segments in the LV [20,29] and RV [18]. Reproducibility is expressed as 95% limits of agreement in the LV [20] and RV [18]

		**TDI**		**2DSE**	
		
		**LV**	**RV**	**LV**	**RV**
**Feasibility**		**85; 93%**	**93%**	**88%**	**93%**

**Reproducibility** Interobserver	Strain (%)	-17.1 ; 28.5	-9.2 ; 9.8	-7.5 ; 9.7	-8.6 ; 11.8
	SR (s^-1^)	-0.76 ; 0.96	-0.91 ; 0.81	-0.77 ; 0.83	-0.79 ; 0.81
**Reproducibility** Intraobserver	Strain (%)	-9.4 ; 9.6	-9.3 ; 9.9	-0.16 ; 2.28	-13.1 ; 15.3
	SR (s^-1^)	-0.42 ; 0.54	-0.85 ; 0.91	-0.54 ; 0.54	-0.73 ; 0.75

## Clinical examples

Clinical and experimental research on the potential role of deformation imaging in various clinical settings is currently an on going topic. The additional value of deformation imaging in echocardiography has been proposed in various pathological conditions although more research is needed [22] In addition, tissue deformation imaging has given us recent new insights into ventricular function, adaptation and mal-adaptation in response to pathology. In the following section we present some clinical examples (case reports) of tissue deformation imaging (either TDI or 2DSE) in various cardiac pathology, underlining the additional advantage in the field of echocardiography.

### Hypertrophic cardiomyopathy

Hypertrophic (obstructive) cardiomyopathy (HCM) is a genetic disorder characterized by hypertrophy of the myocardium. The differentiation of HCM from hypertensive left ventricular hypertrophy (H-LVH) on the basis of morphological information obtained by conventional echocardiography is cumbersome. Myocardial disarray is a typical histopathological finding in HCM and provides a substrate for potential lethal ventricular arrhythmias. This disarray also induces functional abnormalities, which could be detected using tissue deformation imaging. A recent article found that this technique reliably distinguishes HCM from H-LVH with a cut-off value for peak systolic strain of -10.6% [23]

**Figure 11:**
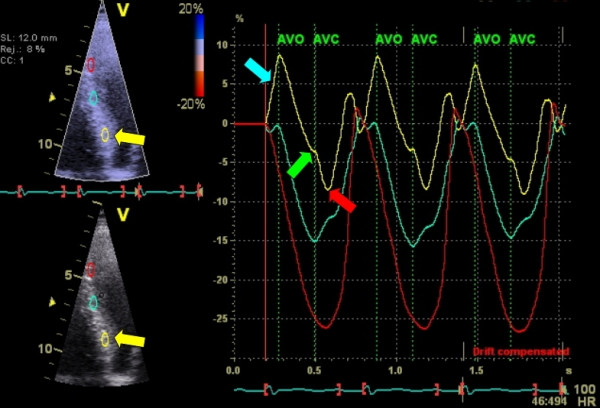
**Tissue Doppler derived strain curve of three cardiac cycles of a patient suspected for HCM**. Yellow-curve/arrow = basal segment, cyan = mid, red = apex. Note the abnormal deformation pattern in the yellow curve. There is prestrech (blue arrow), a reduced peak systolic value (green arrow) and post-systolic shortening (red arrow).

In the following example we present an asymptomatic first degree family member (male, 44 years) of a patient with HCM. This patient had the same mutation as the index patient. On conventional echocardiography a septal thickness of 12 mm (upper limit of normal) was measured with no wall motion abnormalities. Deformation imaging (TDI) unmasked a pathologic deformation in the basal segment of the septum with prestrech, a low peak systolic strain (-3.3%) and post-systolic shortening (Figure 11).

### CRT-patient selection

A potential clinical role of tissue deformation imaging is for patient selection for cardiac resynchronization therapy. The maximal delay between wall segments can be visualized using different parameters. The application of regional deformation imaging in this patient group has the advantage to be able to carefully calculate timing issues of regional deformation properties of the myocardium, independent of translated displacement induced by adjacent or opposite wall segments. In addition, LV ejection fraction can be estimated using global LV end-systolic strain (Figure 2).

**Figure 12:**
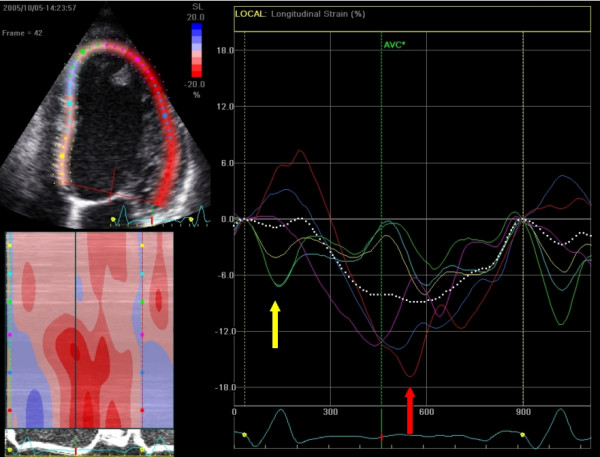
**Longitudinal strain of one cardiac cycle in the 4-chamber view using 2DSE before CRT device implantation**. Note the early septal peak-systolic strain (yellow arrow/graph) simultaneous with lateral stretching and the late lateral peak strain values (red arrow/graph).

**Figure 13:**
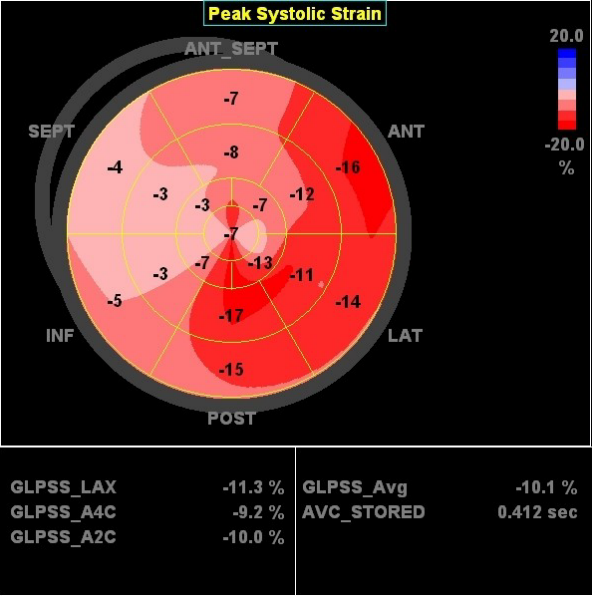
**Bulls-eye figure of peak systolic strain values in the left ventricle before CRT device implantation**. Red = normal strain values, pink = reduced value, light pink = severely reduced values. Global strain of the left ventricle is -10.1%.

**Figure 14:**
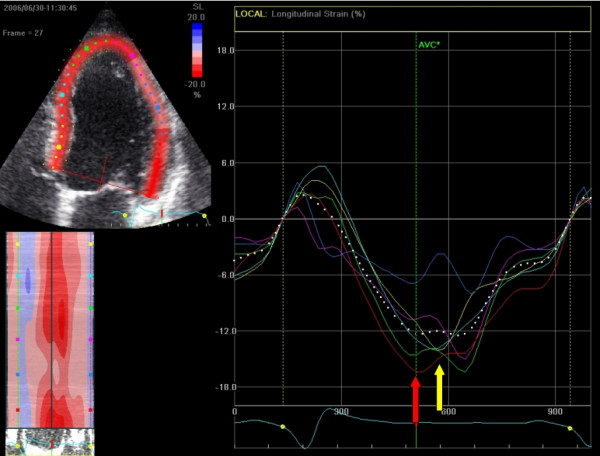
**Longitudinal strain of one cardiac cycle in the 4-chamber view using 2DSE after CRT device implantation**. Note the synchronous contraction pattern of all wall segments and the increase in septal strain to the total ejection. The septal to lateral delay has decreased (distance between yellow and red arrow) after resynchronization therapy. Note the pre-exitation in the mid-lateral wall in combination with mildly reduced strain values, indicating the position of the LV lead.

**Figure 15:**
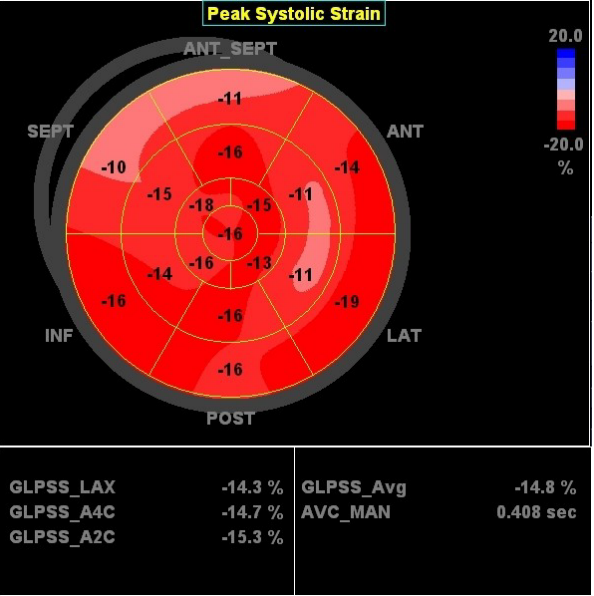
**Bulls-eye figure of peak systolic strain values in the left ventricle after CRT device implantation**. The global systolic strain of the left ventricle has increased to -14.8%. Note the improvement of systolic deformation in the septal wall compared to pre-implantation (see figure 11).

In the following example a patient (male, 54 years) is presented fulfilling CRT criteria (LBBB with a QRS-width > 120 ms, NYHA-class III-IV and an ejection fraction of 22%) who was implanted with a CRT-device. Prior to implantation, we found a septal-to-lateral delay to peak strain value of 425 msec using 2DSE (Figure 12). A bulls-eye plot of peak systolic strain values is presented in figure 13. After 8 months follow up after device implanation, there was a significant improvement of ejection fraction (to 42%) a reduction in LV volume and a dramatic improvement of longitudinal deformation during systole (figure 14). 2DSE revealed a nearly synchronous longitudinal shortening with a septal-to-lateral delay of -60 msec (figure 15).

### Right ventricular function

Although clinical and preclinical research of left ventricular function is broadly being explored, little is known about normal and pathologic right ventricular (RV) function and there is an urgent need for more insight in RV function by quantitative techniques [24] In our own experience, the quantification of regional RV function using either 2DSE or TDI is feasible and reproducible [18] This technique has a potential place in the assessment and follow up of patients with primary (see below) or secondary RV disease such as pulmonary artery disease [25].

**Figure 16:**
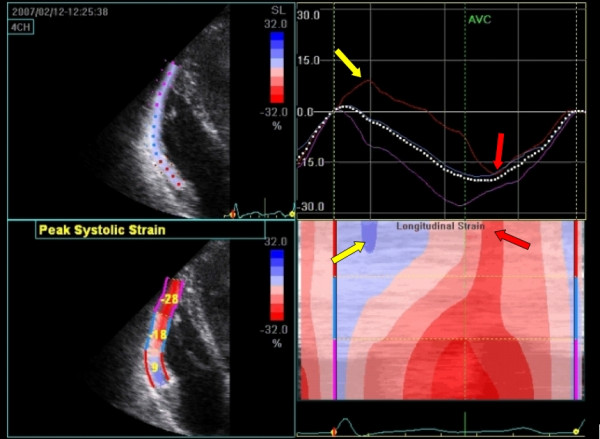
**Deformation imaging of the right ventricle in a patient suspected for ARVD**. 2DSE trace of the right ventricle during one cardiac cycle (top-left). Note the low peak systolic strain (bottom-left) in the basal segment. When visualized graphically (top-right) or with color-map M-mode (bottom-right) the abnormal deformation pattern becomes evident, there is prestretch (blue area on M-mode and positive deflection in graph, yellow arrow) and post-systolic shortening (negative deflection after pulmonic valve closure (AVC) in the graph and late-red area on the color-coded M-mode, red arrow). 2DSE deformation imaging can be seen in the additional file (see Additional File 3).

The following case is a 36 year old woman evaluated for arrhythmogenic right ventricular dysplasia (ARVD), an inherited cardiomyopathy characterized by fibro-fatty myocardial replacement predominantly in the right ventricle. She has a first degree family member diagnosed with ARVD. No genetic defect was found in either. The RV was not dilated, no wall motion abnormalities could be detected nor any other typical ARVD findings on conventional echocardiographic examination [26]. Tissue Doppler strain analysis as well as 2DSE revealed an abnormal deformation pattern in the basal segment (Figure 16/Additional File 3). Based on this finding, this patient scored a minor criterion (regional RV hypokinesia) fulfilling the diagnostic criteria for ARVD (one major and two minor criteria).

## Future perspectives

Several new developments are to be expected, not only to improve tracking quality, reduction noise and improve post processing time [27], but also to make this promising technique more clinically appealing. New automated programs for 2DSE ("automated function imaging"), for instance, allow rapid evaluation of left ventricular deformation in a 17 segment model and visualization of peak systolic strain and post systolic strain index in a bulls-eye figure.

Angle dependency and the impact of artifacts in TDI derived parameters and the low temporal resolution in 2DSE are important limiting factors in the technology. A combination of the two techniques (high quality, angle independent gray scale data and Doppler data with high temporal resolution) could enhance the robustness of the technique and reduce the post-processing time.

MRI-tagging is a technique to calculate deformation in three dimensions. As explained earlier, new echocardiographic techniques have "upgraded" the one-dimensional tissue Doppler to 2DSE. A next step in development is the calculation of strain in three dimensions. With the recent advances in three-dimension echocardiography, this could be possible in the near future. Currently, the low temporal resolution is a severe limitation which has to be overcome before accurate calculations of deformation parameters are feasible.

## Conclusion

Tissue deformation imaging is a rapidly evolving technique able to objectively evaluate regional myocardial function. In this review we aimed to give insight into the basic concepts, image acquisition, data analysis and potential clinical applications. Although this quantification provides both the clinician as well as the researcher with detailed information on the myocardial function during the cardiac cycle, it should be kept in mind that, like all ultrasound techniques, image acquisition and quality remain a limiting factor. Although tissue deformation imaging is a promising technique, knowledge of image acquisition, post processing techniques and interpretation of strain and strain-rate graphs are essential to reliably differentiate myocardial pathology from artifacts both in the clinical setting, and for reliable and reproducible research. Its limitations notwithstanding, tissue deformation imaging is an accurate and robust techniques that may successfully fulfill the need for quantitative assessment of myocardial function. The application of this technique in the clinical setting is currently not routine, but the potential of tissue deformation imaging and the need for quantitative measurements of ventricular function should promote its introduction in various clinical settings in the near future.

## Competing interests

The author(s) declare that they have no competing interests.

## Authors' contributions

AT designed the review, participated to analyzed paper collection and drafted the paper.

BB and PM helped to draft the manuscript (2DSE section and Doppler section respectively).

GS and PD revised the article.

MC helped to draft the manuscript.

All authors read and approved the final manuscript.

## Supplementary Material

Additional file 1 **ROI tracking in tissue Doppler imaging**. The ROI is tracked (bottom left) as described in the text in the basal segment of the RV. Note the changes in the strain-graph during the tracking. After careful tracking, the ROI follows the myocardial motion. Click here for file

Additional file 2 **Impact of stationary artifact on 2DSE**. Appropriate ROI in a 4-chamber view using 2DSE despite a prominent stationary artifact in the lateral wall. The ROI is color coded: yellow-red = negative SR, green = no SR and blue = positive SR. In the region of the artifact there is no reversal of SR values, all myocardial segments show identical color coding during each phase of the cardiac cycle. See figure 9 for additional information. Click here for file

Additional file 3 **Deformation imaging of the right ventricle in a patient suspected for ARVD using 2DSE**. 2DSE parameter extraction in the right ventricle. A ROI is tracked along the RV free wall and follows its movement during the cardiac cycle. An overlying color map is seen on the ROI: red indicates normal deformation; pink indicates reduced deformation and blue indicates no deformation, or stretch. See figure 16 for additional information. Click here for file
